# Neuron–Glioma Synapses in Tumor Progression

**DOI:** 10.3390/biomedicines14010072

**Published:** 2025-12-29

**Authors:** Cristina Cueto-Ureña, María Jesús Ramírez-Expósito, José Manuel Martínez-Martos

**Affiliations:** Experimental and Clinical Physiopathology Research Group CTS-1039, Department of Health Sciences, School of Health Sciences, University of Jaén, E23071 Jaén, Spain; ccueto@ujaen.es (C.C.-U.); mramirez@ujaen.es (M.J.R.-E.)

**Keywords:** gliomas, neuron–glioma synapses, tumor microtubules, glutamate, GABA, tumor proliferation, therapy resistance

## Abstract

Gliomas are the most common malignant primary brain tumors in adults. The treatment of high-grade gliomas is very limited due to their diffuse infiltration, high plasticity, and resistance to conventional therapies. Although they were long considered passive massive lesions, they are now regarded as functionally integrated components of neural circuits, as they form authentic electrochemical synapses with neurons. This allows them to mimic neuronal activity to drive tumor growth and invasion. Ultrastructural studies show presynaptic vesicles in neurons and postsynaptic densities in glioma cell membranes, while electrophysiological recordings detect postsynaptic currents in tumor cells. Tumor microtubules (TMs), dynamic cytoplasmic protrusions enriched in AMPA receptors, are the structures responsible for glioma–glioma and glioma–neuron connectivity, also contributing to treatment resistance and tumor network integration. In these connections, neurons release glutamate that mainly activates their AMPA receptors in glioma cells, while gliomas release excess glutamate, causing excitotoxicity, altering the local excitatory-inhibitory balance, and promoting a hyperexcitable and pro-tumorigenic microenvironment. In addition, certain gliomas, such as diffuse midline gliomas, have altered chloride homeostasis, which makes GABAergic signaling depolarizing and growth promoting. Synaptogenic factors, such as neuroligin-3 and BDNF, further enhance glioma proliferation and synapse formation. These synaptic and paracrine interactions contribute to cognitive impairment, epileptogenesis, and resistance to surgical and pharmacological interventions. High functional connectivity within gliomas correlates with shorter patient survival. Therapies such as AMPA receptor antagonists (perampanel), glutamate release modulators (riluzole or sulfasalazine), and chloride cotransporter inhibitors (NKCC1 blockers) aim to improve outcomes for patients.

## 1. Introduction

Gliomas represent the most frequent malignant brain tumors in adults [1,2,3,4] and constitute one of the leading causes of neurological impairment [1]. High-grade gliomas are characterized by their unfavorable prognosis and treatment-resistant nature [4,5]. Specifically, glioblastoma (GBM), the highest grade type of glioma according to the World Health Organization (grade IV), has a median survival of approximately 16 months and almost universal lethality [2,4]. Despite research efforts, survival in high-grade gliomas has not improved significantly over the past two decades [6]. Recurrence is almost inevitable and often occurs outside surgical margins [7], in part because neuron-to-glioma synapses confer resistance to both surgery and chemotherapy [3,5,8,9].

The traditional understanding of gliomas was limited to viewing them as destructive entities that replaced and destroyed healthy brain tissue, causing loss of function by physically occupying brain space [1,10]. However, this point of view has evolved after recent research, which has revealed a more complex reality. Thus, current studies demonstrate that glioma cells not only passively coexist in brain tissue but also establish active and dynamic interactions with surrounding neurons [1,2,6,10,11,12]. Nervous system activity has emerged as a driver of the progression and initiation of primary brain cancers [10,13,14].

**Figure 1:**
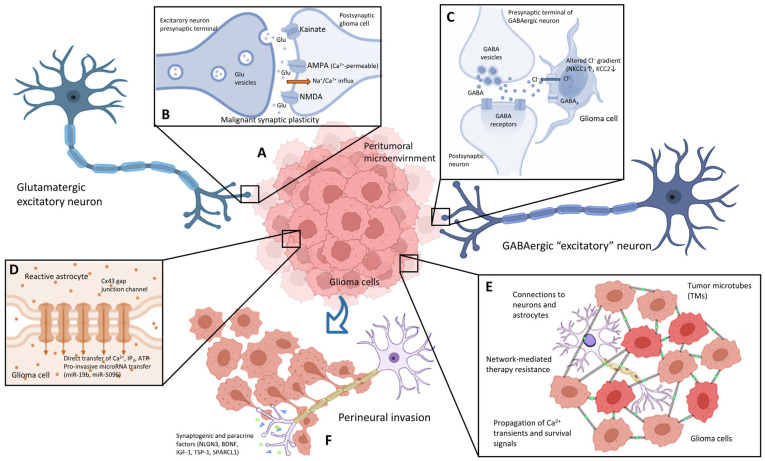
Integration of glioma cells into neural circuits and main neuro-tumor communication pathways. (**A**) A cluster of glioma cells infiltrating the brain parenchyma is shown, surrounded by neurons and glial cells that make up the characteristic tumor microenvironment. (**B**) The glutamatergic neuron–glioma synapse promotes the release of glutamate, which acts on calcium-permeable AMPA/NMDA receptors located in the tumor cell membrane and, preferentially, in tumor microtubules, generating excitatory postsynaptic currents and depolarization of the glioma cell. (**C**) Multiple modulatory synaptic contacts include GABAergic synapses, which in subtypes such as diffuse midline gliomas (DMGs), due to the overexpression of Na^+^-K^+^-2Cl^−^ cotransporter 1 (NKCC1) and the decrease in KCC2, acquire a depolarizing character and promote tumor proliferation and circuit hyperexcitability. (**D**) Intercellular coupling via gap junctions, mainly mediated by Cx43, allows the direct passage of ions and second messengers such as Ca^2+^, ATP, IP3, and microRNAs between tumor and astroglial cells, facilitating calcium wave propagation, invasion, and resistance to therapies. (**E**,**F**) Tumor microtubules (TMs) appear as ultra-long extensions that connect glioma cells to each other and to neurons and astroglia, acting as cables that redistribute organelles, coordinate network responses, channel synaptic inputs, and confer plasticity and relative insensitivity to radiation and chemotherapy. The network organization of tumor cells and surrounding cells favors the formation of new neuron–glioma synapses, the reinforcement of existing connections, the expansion of TMs, and the establishment of a hyperexcitable and pro-tumorigenic microenvironment, favored by multiple synaptogenic and paracrine factors such as neuroligin 3, brain-derived neurotrophic factor/tropomyosin receptor kinase B (BDNF/TrkB), insulin-like growth factor 1 (IGF-1), thrombospondin-1 (TSP 1), and secreted protein acidic and rich in cysteine like 1 (also known as hevin) (SPARCL1). Also, perineural invasion occurs. Gliomas are therefore active elements of neuronal circuits, capable of exploiting synaptic plasticity and glioneuronal communication mechanisms to drive their own proliferation, invasion, circuit remodeling, and therapeutic resistance.

These interactions go beyond simple paracrine chemical signaling, including the formation of direct electrochemical synapses between neurons and tumor cells. The discovery of these synaptic connections has transformed the understanding of brain cancer [1,2,10,11,15,16,17]. Therefore, glioma cells can functionally integrate into existing neural networks, modifying the electrical activity of the brain and altering the way the infiltrated regions process information [1,3,5,10,14,18]. This synaptic integration allows tumors to not only survive in the brain environment but also to actively use neuronal activity as fuel for their growth and spread [2,3,4,5,6,7,8,9,10,11,13,14,19,20,21,22,23,24,25,26,27,28,29,30,31,32,33] (Figure 1).

## 2. Evolution of the Glioma Concept

The knowledge about gliomas has undergone a transformation reflecting technological and conceptual advances in neuroscience and oncology [1,2,3,5,8,10,11,12,19,26]. Initially, the medical perception of these tumors was based on the concept of the “mass effect,” where neurological symptoms were believed to result solely from the physical compression and destruction of healthy brain tissue by the tumor mass [1,10,11]. This perspective, although partially correct, was insufficient to explain the complexity of neurological symptoms observed in patients.

Many individuals with gliomas had seizures, subtle cognitive alterations, and personality changes that did not directly correlate with tumor size or location [1,4,11,22,26,31,34]. These clinical findings suggested the existence of additional, more sophisticated mechanisms. The development of advanced neurophysiological techniques and the ability to record brain electrical activity in real time, such as electrocorticography (ECoG), has clearly demonstrated them [1,3,22]. Brain regions infiltrated by gliomas showed altered electrical activity patterns that could not be explained solely by the physical presence of the tumor, including synchronous activity with a diffuse spatial network and decreased neuronal signaling [1,3].

Definitive evidence of direct synaptic interactions between neurons and glioma cells emerged with high-resolution electron microscopy and electrophysiological techniques [35,36]. These studies revealed the presence of true synaptic structures, with presynaptic vesicles, clearly defined synaptic clefts, and postsynaptic densities in tumor cell membranes [19,22,26,29]. Thus, the emerging field of “cancer neuroscience” examines the bidirectional interactions between the nervous system and cancer cells [1,2,3,4,5,8,10,11,12,13,15,16,25,26,28,37]. This new perspective recognizes the brain not as a passive tumor host but also as an active participant in glioma pathogenesis, where normal neuronal activity can be co-opted to promote tumor growth and invasion [1,2,3,5,6,7,8,10,11,12,15,16,26,28].

Multiple clinical observations have documented that gliomas occur more frequently in brain regions with high activity [3,5] and that brain regions infiltrated by tumors remodel functional neural circuits [22] and activate a more diffuse spatial network during cognitive tasks [1,3]. These findings also correlate negatively with patient survival and performance on cognitive tasks [1,3,22], suggesting a direct connection between neuronal activity and tumor progression, where glioma cells may even acquire neuronal characteristics to facilitate invasion [10,38,39,40,41,42].

## 3. Glioma Biology and Tumor Microenvironment Characteristics

Gliomas constitute a heterogeneous family of tumors that originate from glial cells of the central nervous system (CNS), accounting for approximately 50–80% of all primary malignant brain tumors [1,2,5,11,24,43]. GBM is the most aggressive and common form in adults, showing high invasiveness [2,4,8,9,10,19,20,24] and resistance to treatments [5,7,11,23,24]. Unlike other tumors that form compact masses, glioma cells migrate extensively throughout the brain parenchyma, infiltrating apparently normal tissue at considerable distances from the main tumor, even to distant structures such as the brainstem (Figure 1). This invasive property makes complete surgical resection virtually impossible and contributes significantly to the high recurrence rate [1,3,5,7,10,11].

The aggressive behavior of gliomas is linked to the characteristics of their tumor microenvironment (TME) [2,43,44,45,46,47]. This ecosystem includes not only cancer cells but also a diversity of non-malignant cells, including neurons, astrocytes, microglia, macrophages, endothelial cells, and pericytes [2,4,10,11,48]. All these cell populations establish a bidirectional communication network that influences tumor behavior [1,2,3,4,5,6,8,10,11,12,32].

An important structural component of the glioma microenvironment are tumor microtubules (TMs), whose presence has been observed in all types of incurable glioma studies. TMs are specialized cytoplasmic structures that connect glioma cells to each other and to other cells in the microenvironment [2,3,5,8,22,49]. TMs can extend for considerable distances [8], allowing direct communication between tumor cells through connexin 43 (Cx43)-mediated gap junctions stabilized by p120 catenin, forming a functional syncytium capable of transporting small molecules such as calcium ions, ATP, IP3, and microRNAs [4] and signals such as calcium waves. They also allow the redistribution of organelles such as mitochondria and nuclei [2,3,5,8,11,22,26].

This network of connections facilitates the coordination of tumor behavior and confers resistance to conventional therapies, including chemotherapy and radiotherapy, by enabling the distribution of toxic metabolites and cellular repair [3,5,8,10,21]. TMs can also establish contacts with non-tumor cells, including astrocytes and neurons [5], integrating tumor cells into the existing neural and glial network [1,2,3,4,6,8,9,10,11,12,18,19,22,23,24,27,28,29,30,50] and expanding the scope of tumor influence beyond the physical boundaries of the cancer cells themselves [1,3]. The formation and dynamics of these microtubules are regulated by synaptic activity, creating a mechanism by which neuronal stimulation can directly promote tumor connectivity and growth [2,3,5,6,9,10,12,13,14,18,20,22,23,24,27,28,29,30,49]. The presence of TMs is a feature observed in all types of incurable glioma studies so far [10].

Cellular heterogeneity represents another important aspect of glioma biology. Multiple cancer cell subpopulations with distinct genetic, epigenetic, and phenotypic characteristics coexist within the same tumor [5,13,18,19,20,22,29,30,43,47,51,52,53,54]. This intratumoral diversity allows different glioma regions to respond variably to selective microenvironment pressures, including nutrient availability, oxygen levels (hypoxia) [10,18], and treatment exposure [3,5,18].

A particularly important population within this heterogeneity are glioma stem cells (GSCs) [10,22,51,55,56], which possess self-renewal and multilineage differentiation capacity. These cells are considered responsible for glioma initiation, maintenance, and recurrence. Moreover, they show remarkable resistance to conventional therapies, contributing to therapeutic failure [2,4,10,11,55,56].

The vascular microenvironment of gliomas also exhibits unique features [2,4,10]. High-grade gliomas are highly angiogenic, promoting the formation of an aberrant vasculature [11,23,57]. This pathological neovascularization not only supplies nutrients to the growing tumor but also facilitates the dissemination of tumor cells to distant sites [3,7,10,11,57].

## 4. Structural and Functional Evidence for Neuron–Glioma Synapses

The demonstration of the existence of authentic synapses between neurons and glioma cells has required the use of multiple complementary methodological approaches, from ultrastructural analyses to real-time electrophysiological recordings [11,12]. Therefore, these connections are not experimental artifacts but functional interactions with significant biological relevance, supporting the notion that tumor infiltration simply leads to destruction and loss of function [1,10].

Ultrastructural evidence has revealed in neuron–glioma synapses distinctive morphological features of functional connections. Electron microscopy analyses have identified the presence of clustered synaptic vesicles at neuronal presynaptic terminals, clearly defined synaptic clefts, and specialized postsynaptic densities in glioma cell membranes, which act as the postsynaptic partner [12]. These synaptic structures have been described both in experimental animal models (such as mouse xenografts [12,19,58,59] and *Drosophila* models [10,24]) and in human tissue obtained from glioblastoma and astrocytoma patients [27]. The ability of cancer cells of different lineages to use the same language as native neurons and the conservation of these features across species suggest that neuron–glioma synapses represent an evolutionarily conserved mechanism of interaction between tumor cells and the nervous system [24].

Immunofluorescence has complemented ultrastructural studies by visualizing the colocalization of specific synaptic markers at neuron–glioma interfaces. Presynaptic markers such as Synapsin-1 [19,22] and postsynaptic markers such as PSD95 [22,60] are found to be appropriately distributed, confirming their synaptic identity. It is particularly remarkable that neuron–glioma synapses can form not only on the cell bodies of tumor cells but also on their TMs [3,5]. This widespread distribution suggests that glioma cells may receive multiple synaptic inputs along their extensions, potentially maximizing their ability to integrate neuronal signals [1,3,5,10,22,23].

**Figure 2:**
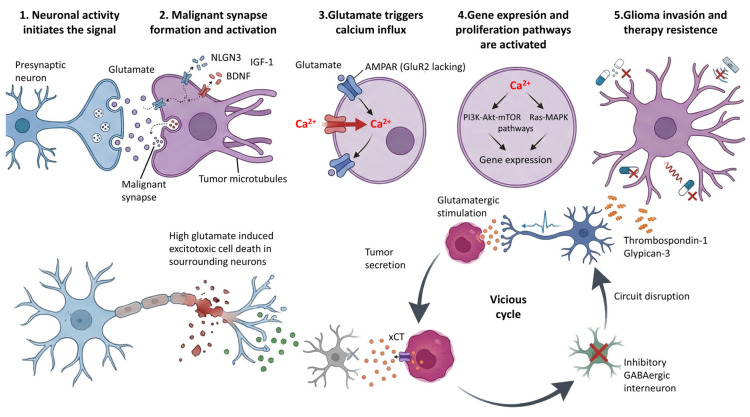
Functional synaptic connections between neurons and glioma cells fuel tumor progression. Excitatory projection neurons form functional glutamatergic synapses directly onto glioma cells in the infiltrative margin, enabling action-potential-dependent glutamate release into the synaptic cleft. Postsynaptic glioma cells express calcium-permeable AMPA-type glutamate receptors with under-edited or low GluA2, so neuronal firing evokes large excitatory postsynaptic currents and membrane depolarization in the tumor cell. Activity-regulated paracrine factors such as neuroligin-3 (NLGN3), brain-derived neurotrophic factor (BDNF) and insulin-like growth factor-1 (IGF-1) further potentiate synaptogenesis and strengthen synaptic input. Downstream of synaptic depolarization and Ca^2+^ influx, oncogenic signaling cascades including PI3K-Akt-mTOR, Ras-MAPK and other voltage-sensitive pathways are activated, promoting glioma cell proliferation, survival and migration. Tumor cells remodel surrounding neural circuits through glutamate release, secretion of synaptogenic factors such as thrombospondin-1 and glypican-3, and loss of inhibitory interneurons, thereby driving neuronal hyperexcitability, seizures and increased functional connectivity of tumor-infiltrated cortex. This reciprocal neuron–glioma network establishes a feed-forward loop in which neuronal activity accelerates tumor growth and invasion, while the tumor further amplifies pathological network activity.

Electrophysiological evidence has also provided functional proof that neuron–glioma synapses are electrically active and capable of transmitting information. Whole-cell patch-clamp recordings have shown that glioma cells can generate excitatory postsynaptic currents (EPSCs) in response to neuronal stimulation. These EPSCs occur on a millisecond scale [12,29,30] (Figure 2). These postsynaptic currents are mediated primarily by AMPA (α-amino-3-hydroxy-5-methyl-4-isoxazole propionic acid) receptors [3,4,5,6,8,9,10,11,12,21,23,26,29,30]. These receptors preferentially localize on TMs of glioma cells [8,9,21]. A key aspect is that AMPARs in glioma cells often lack or have low expression of the GluR2 subunit, making them Ca^2+^ permeable [4,26]. Although the involvement of NMDA (N-methyl-D-aspartate) receptors has also been documented in certain contexts [2,4,10,26], studies confirm that glutamatergic currents mediated by AMPARs in glioma cells exhibit electrophysiological features consistent with synaptic transmission [6].

An intriguing aspect is the observation that GABAergic signaling can have paradoxical effects in certain types of glioma (Figure 1). In diffuse midline gliomas (DMGs), activation of GABA receptors results in depolarization rather than hyperpolarization [2,4,19,26]. This reversal of the chloride gradient is due to an elevated intracellular Cl- concentration in DMGs cells, maintained by the function of the Na^+^-K^+^-2Cl^−^ cotransporter NKCC1 [2,4,19,26]. This GABA-mediated depolarization of DMGs cells promotes their proliferation in vivo [19].

Intracellular calcium recordings have shown that synaptic activation of glioma cells induces calcium transients. These changes in intracellular calcium are functionally important because they activate signaling cascades that promote glioma cell proliferation, survival, and invasion. Furthermore, these calcium transients can propagate through the glioma network via gap junctions of MTs [2,5,8,12,14,18,26,29,30,49]. Electrophysiology is useful for directly investigating synaptic responses and membrane potential changes, while calcium imaging is valuable for studying network-level changes [12].

ECoG studies in human patients have also demonstrated that glioma-infiltrated brain regions, despite the presence of the tumor, maintain the ability to engage in synchronized neuronal activity during the performance of cognitive tasks [1]. This ability is observed with coordinated responses in the high gamma range (70 to 170 Hz), a measure that is directly related to the activity of local neuronal populations. It has even been documented that glioma-infiltrated cortex exhibits neuronal hyperexcitability, manifested by an increase in high-gamma band power during speech production. However, these regions show altered activation patterns, suggesting a fundamental modification in the way these networks process information [1]. These patterns include a diffuse recruitment of brain areas. In tasks such as speech planning, while normal cortex activates canonical and spatially restricted areas, glioma-infiltrated cortex recruits a more diffuse spatial network, including atypical areas such as the middle frontal gyrus [1]. This broader distribution of activity could represent an inherently diffuse and interconnected neuronal-glioma network activation [1].

Decreased entropy of temporal signals also occurs. Neuronal signals from glioma-infiltrated cortex exhibit decreased entropy, which is interpreted as a decreased ability to encode information over time [1]. This reduction in the statistical variability of the signals suggests a degradation in the quantity and quality of the encoded information. This decrease in entropy is observed even in brain regions essential for speech production and does not vary significantly according to the 1p/19q codeletion status of the tumor [1].

These functional alterations suggest that although neuron–glioma synapses can integrate into existing neural networks, their presence fundamentally changes the way these networks process information [1]. The decrease in signal entropy may reflect a loss of normal computational complexity in the brain, which could contribute to cognitive deficits observed in glioma patients. It is postulated that this lower entropy could be due to glioma synaptic signaling biasing the activity of these neural networks at baseline [1]. In fact, glioma-infiltrated cortex, unlike normal cortex, fails to decode subtle aspects of speech, such as the distinction between monosyllabic and polysyllabic words [1].

The resulting cognitive deficits significantly affect patients’ quality of life [4,18,26]. These may include impairments in working memory, executive function, attention, and language. Aphasia (speech impairment) is a particularly debilitating and frequent complication in patients with gliomas located in language areas [1,3]. This can result from both direct tissue destruction by the tumor and disruption of neuronal circuitry by aberrant tumor activity [1,23]. Decreased entropy of glioma-infiltrated cortex, even in non-aphasic patients, may be indicative of alterations in cognitive processing that, upon further deterioration, may lead to aphasia [1].

Studies in animal models have confirmed that glioma cells can maintain activity-dependent connections with disparate brain regions. This extended connectivity suggests that tumor cells can receive and integrate information from multiple neural sources, potentially allowing them to respond to diverse types of brain activity [10,22,27,29,30,37].

## 5. Molecular Mechanisms of Synaptic Communication

### 5.1. Glutamatergic Neurotransmission

Glutamate is recognized as the central neurotransmitter in the complex interactions between neurons and gliomas (Table 1). It plays a dual role in glioma biology, acting as the main excitatory neurotransmitter in the central nervous system. On the one hand, neurons release glutamate at neuron–glioma synapses, where it activates AMPA and NMDA receptors on tumor cells. Functional glutamatergic synapses between presynaptic neurons and postsynaptic glioma cells have been documented in both pediatric and adult high-grade gliomas [19,23]. Specifically, NBQX-sensitive glutamatergic synaptic currents have been observed in a subpopulation of DMG cells [19].

**Figure 3:**
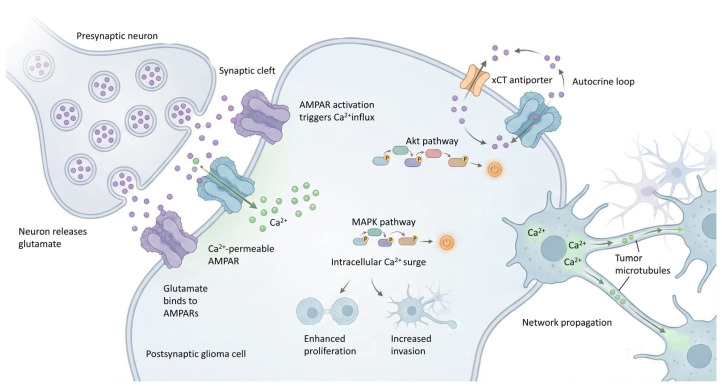
Glutamatergic neuron–glioma synapses and autocrine glutamate release drive intracellular signaling and multicellular network behavior in diffuse gliomas. Presynaptic excitatory neurons release glutamate into the synaptic cleft in response to action potentials, which binds to Ca^2+^-permeable AMPA receptors enriched in GluA1/GluA4 and deficient in edited GluA2 on the postsynaptic glioma membrane. AMPAR opening permits a surge of Na^+^ and Ca^2+^ entry, leading to robust membrane depolarization and elevation of cytosolic Ca^2+^ that acts as a second messenger to activate pro-survival and pro-proliferative pathways including PI3K-Akt and Ras-ERK/MAPK cascades. These signaling events enhance cell-cycle progression, resistance to apoptosis and motility, thereby increasing glioma proliferation and invasion into surrounding brain tissue. Glioma cells also export glutamate via the cystine-glutamate antiporter xCT (SLC7A11), creating an autocrine/paracrine loop that tonically activates tumor AMPARs and contributes to excitotoxic injury and hyperexcitability in neighboring neurons. Elevated intracellular Ca^2+^ is transmitted across an interconnected network of glioma cells coupled by tumor microtubes and gap junctions containing connexin-43, enabling synchronized Ca^2+^ waves, coordinated invasion fronts and multicellular resistance to chemo- and radiotherapy. Together, this circuitry illustrates how the glutamate-AMPAR axis integrates synaptic input, metabolic exchange and tumor-intrinsic wiring to fuel glioma growth, dissemination and treatment resistance.

On the other hand, glioma cells themselves secrete large amounts of glutamate into the extracellular space, mainly through the cystine-glutamate antiporter system (xCT or SLC7A11 system) [34]. This non-synaptic release of glutamate by glioma cells may drive glioma invasion [8,10,61,62]. This massive release of glutamate by tumor cells generates a pathological positive feedback loop that facilitates glutathione synthesis, which in turn mitigates oxidative stress and sustains glioblastoma cell survival [8]. Elevated levels of glutamate in the tumor microenvironment not only stimulate the growth of glioma cells by activating their own receptors but also induce excitotoxicity in the surrounding neurons [3,4,5,6,8,9,10,11,12,21,23,26,29,30]. This excitotoxicity is particularly harmful to inhibitory GABAergic interneurons, causing their selective death. The death of these peritumoral inhibitory neurons alters the local excitatory–inhibitory balance, which promotes neuronal hyperexcitability and tumor progression (Figure 3) [11,62].

AMPA receptors have been observed to be highly expressed in high-grade gliomas. These cells exhibit pharmacological and kinetic properties similar to their neuronal counterparts. However, their activation in the tumor context triggers specific oncogenic signaling cascades. Stimulation of these receptors not only depolarizes glioma cells but also activates kinase pathways, including MAPK and Akt pathways, that promote glioma cell proliferation, survival, motility, and invasion [6,29,30,32,50].

Additionally, the PI3K/Akt/mTOR pathway is frequently activated in glioblastoma, often due to various mutations or loss of phosphatase and tensin homolog (PTEN) protein [4]. These processes inherently require biomolecule synthesis and cell division to sustain rapid tumor growth. In this way, pharmacological or genetic blockade of AMPA receptors can reduce the proliferation and invasion of glioma cells [5,63]. Perampanel, a noncompetitive AMPA receptor antagonist [4,21,31,64], is being investigated in clinical trials for brain tumor-related epilepsy [9,31,65,66,67,68,69,70]. These receptors predominantly localize to TMs [71,72,73], and AMPA receptor-mediated neuronal activity drives the formation, elongation, and dynamics of TMs, which are essential for brain colonization of glioblastoma cells. BDNF-TrkB (brain-derived neurotrophic factor-receptor tyrosine kinase B) signaling is crucial, as it promotes AMPA receptor trafficking to the postsynaptic membrane of glioma cells [30]. This results in an increase in the amplitude of glutamate-evoked currents and enhances malignant synaptic plasticity [30].

Ablation of BDNF-TrkB signaling inhibits tumor progression [30]. BDNF is essential for hippocampal long-term potentiation (LTP) [30]. Furthermore, neuroligin-3 (NLGN3) released by neurons in response to activity drives glioma progression [6,27,30,74,75,76]. NLGN3 expression correlates with survival in glioblastoma patients [6], and its signaling activates pathways such as Akt, Erk, and mTORC1 in glioma cells [32]. Knockdown of NLGN3 in glioma models reduces tumor proliferation [27,29,30].

The fact that neuronal activity promotes glioma growth through a complex interplay of converging mechanisms [6,9,10,21], allows the integration of tumor cells into the brain parenchyma, establishes connections with neurons, alters brain functionality, and accelerates cancer progression [10], supports a bidirectional interaction between the nervous system and cancer that would allow the development of new therapeutic strategies [20]. In this sense, evidence indicates that blockade of Ca2+-permeable AMPA receptors suppresses migration and induces apoptosis in human glioblastoma cells, suggesting that their activation promotes these malignant processes [5,77]. Antiepileptic drugs, such as perampanel, an AMPA (glutamatergic) receptor antagonist, have also been studied in glioblastoma.

Glioblastomas also express markers of synaptogenesis, including kainate (GRIK2) and NMDA (GRIN2C) receptor genes [20]. Data suggest advanced neuronal differentiation in glioma cells, with synapse-like structures forming direct non-functional cell–cell contacts between glioma cells [34]. Studies in *Drosophila* have shown that presynaptic genes such as *brp* (active zone factor), *Lip-α* (Liprin-alpha), and *syt1* (synaptotagmin 1) are required for glioblastoma progression [24,78]. Silencing of *syt1* or *syt4* in glioblastoma cells prevents tumor expansion and reduces cell number. Silencing of *brp* or *lip-α* also reduces tumor microtubule volume. In addition, glutamate receptors (GluR) are required for tumor volume expansion and growth (via cytonemes/TMs), although not for cell number increase. The reduction in calcium influx into glioblastoma cells following presynaptic and postsynaptic gene silencing suggests a functional contribution of these synaptic genes to calcium influx into tumor cells [24,78].

**Table 1:** Neurotransmitter mechanisms in glioma progression.

Mechanism/ Pathway	Description	Effect on Glioma or Microenvironment	Key Molecules, Receptors or Transporters	Pathological Outcome Relevant Glioma Type	Therapeutic Target
Glutamatergic synapse [4,63,71,72,73]	Neurons release glutamate at the neuron–glioma synapse, activating AMPA and NMDA receptors on tumor cells. Promotes depolarization and Ca^2+^ influx, triggering oncogenic signaling (MAPK, Akt, PI3K/Akt/mTOR).	EPSCs in glioma cells. AMPAR activation drives TMs formation and dynamics. Overall, promotes proliferation, invasion, and increased malignant plasticity.	AMPARs, often Ca^2+^ permeable due to lack of GluR2; NMDARs; TMs.	High-grade gliomas. Proliferation, invasion, TMs formation, increased malignant plasticity.	Perampanel AMPA antagonist. Disruption of TMsMemantine NMDA antagonist.
Autocrine or paracrine glutamate release [4,63,71,72,73]	Glioma cells secrete large amounts of glutamate into the extracellular space in an autocrine/paracrine manner.	Extracellular glutamate levels increased ~100-fold. Induces excitotoxicity in surrounding inhibitory GABAergic interneurons, shifting the excitatory-inhibitory balance, promoting neuronal hyperexcitability and glioma progression and invasion.	Cystine-glutamate antiporter xCT (SLC7A11 system).	General glioma/GBM. Promotes tumor growth, neuronal hyperexcitability, excitotoxicity, and therapeutic failure.	Sulfasalazine (xCT inhibitor). Riluzole/troriluzole (modulators of glutamate release).
GABAergic excitation [4,13,29,30,79]	Activation of GABA_A_ receptors in tumor cells results in depolarization instead of inhibition due to high intracellular Cl^−^.	GABA-mediated depolarization caused by reversal of the chloride gradient. Promotes proliferation and tumor growth in DMGs, contributing to network hyperexcitability.	GABA_A_Rs; Na^+^-K^+^-2Cl^−^ cotransporter NKCC1 (overexpressed); KCC2 (reduced).	DMGs. Proliferation and hyperexcitability.	Bumetanide (NKCC1 inhibitor). GABA receptor inhibitors (e.g., flumazenil).
Synaptogenic paracrine factors [13,29,30,71,72,73,79]	Tumor and/or neuronal cells release synaptogenic factors that enhance neuron–glioma synaptogenesis and plasticity.	Neuroligin-3 (NLGN3) activates PI3K-mTOR signaling and promotes proliferation. BDNF enhances AMPAR trafficking and synaptic plasticity. TSP-1 and SPARCL1 promote synapse formation, increasing synaptogenesis, malignant plasticity, and tumor invasion.	Neuroligin-3 (NLGN3); BDNF/TrkB; Thrombospondin-1 (TSP-1); SPARCL1; α2δ-1 subunit.	Proliferation, synaptogenesis, malignant plasticity, increased tumor invasion.	TrkB inhibitors (genetic/pharmacological). Gabapentin/Pregabalin (α2δ-1 binders).
Structural connectivity/Tumor network [29,30,63,71,72,73,79]	Glioma cells form multicellular networks via tumor microtubules and gap junctions, enabling structural and functional connectivity.	Propagation of Ca^2+^ waves across the tumor network, distribution of organelles and toxic metabolites among connected cells. Facilitates coordinated growth, invasion, and resistance to chemo- and radiotherapy.	TMs; Connexin 43 (Cx43) Gap Junctions.	Therapeutic resistance (chemo/radio), invasion, coordinated growth, increased malignant plasticity.	Gap junction inhibitors (e.g., meclofenamate). Agents disrupting TMs (e.g., Perampanel).
Cholinergic signaling [13,29,30,71,72,73,79]	Cholinergic neurons signal to DMG cells primarily through muscarinic receptors M1 and M3. DMG cells in turn enhance cholinergic circuit activity.	Cholinergic neuronal activity promotes DMG proliferation, Ca^2+^ transients, migration, and circuit-dependent growth. DMGs reciprocally increase cholinergic activity, creating a feed-forward loop between tumor and neuronal network.	Muscarinic receptors M1 (CHRM1) and M3 (CHRM3).	DMGs. Promotes proliferation, migration, and circuit-dependent growth.	M1/M3 receptor antagonists (e.g., VU0255035, 4-DAMP).
Dopaminergic signaling [4,29,30,71,72,73,79]	Dopamine signaling via DRD2 in GBM cells activates oncogenic pathways and modulates susceptibility to apoptosis-inducing ligands.	DRD2 activates MET signaling, supporting GBM stemness and clonogenic growth. DRD2 inhibition promotes interaction of MET with TRAIL receptors (DR4/5), sensitizing cells to apoptosis and reducing stem-like properties.	Dopamine receptor D2 (DRD2); MET receptor; TRAIL receptors DR4/5.	Promotes GBM stemness and clonogenic growth via DRD2-MET axis. DRD2 antagonists can induce apoptosis and reduce stem-like features.	DRD2 antagonists (e.g., Perphenazine, ONC201, ONC206).
Acid sensing [4,13,80]	Acidic tumor microenvironment activates neuronal ASIC1a channels, modulating neuronal activity and neurotransmitter release that impact glioma behavior.	Neuronal activation and neurotransmitter release induced by acidic TME support tumor growth. Genetic deletion or pharmacologic inhibition of neuronal ASIC1a reduces tumor size and prolongs survival in preclinical models.	Neuronal acid-sensing ion channel 1a (ASIC1a).	Tumor growth supported by acid-sensing-driven neuronal activity. Deletion or inhibition of ASIC1a reduces tumor burden and prolongs survival.	Pharmacological inhibition of neuronal ASIC1a.

#### Modulation of Glutamate Metabolism

Strategies aimed at reducing pathological glutamate levels in the tumor microenvironment represent a promising therapeutic approach for gliomas [8,26,81]. Glioma cells synthesize and secrete large amounts of this excitatory neurotransmitter [3,81], with vesicular glutamate transporters (VGLUTs) being responsible for incorporating glutamate into synaptic vesicles, with VGLUT1 and VGLUT2 showing complementary distributions in the central nervous system [82]. After glutamate release, its extracellular concentration is increased up to 100-fold in gliomas and their vicinity in human and murine models [11]. In addition, peritumoral reactive astrocytes show an impaired ability to uptake glutamate, resulting in more elevated and toxic levels of glutamate [3,8]. This cycle enhances tumor invasion and neuronal hyperexcitability, amplifying the activity of neuroglial networks [8,62]. Thus, several pharmacological strategies are under investigation to modulate glutamate metabolism.

Sulfasalazine, an anti-inflammatory drug approved for other indications, inhibits glutamate release through the xCT antiporter system [4,8,11,25]. This inhibition significantly reduces extracellular glutamate levels in the tumor microenvironment, which decreases autocrine and paracrine stimulation of tumor growth and alleviates excitotoxicity in surrounding neurons, potentially reducing the incidence of seizures [4,8,11,25]. In animal models, blockade of xCT by sulfasalazine has been shown to reduce tumor growth and improve survival [11]. Sulfasalazine is one of the drugs evaluated in the GLUGLIO clinical trial, a phase Ib/II trial combining it with gabapentin and memantine and standard chemoradiotherapy in patients with newly diagnosed glioblastoma [8,25]. However, a small phase I study of sulfasalazine monotherapy in advanced glioblastoma was terminated for lack of efficacy [25].

Memantine, a noncompetitive NMDA receptor antagonist approved for the treatment of dementia [8,25,26], is under investigation for its potential antitumor effects. Memantine could prevent the formation of synapses between neurons and glioma cells, which would reduce tumor cell invasion and neuroglial signaling [8,25,81]. NMDA receptor antagonism increases radiosensitivity [26]. Its neuroprotective effects may also improve neurocognitive function. Memantine is also part of the GLUGLIO clinical trial [8,25]. Although well tolerated in an early clinical trial, exploratory efficacy results are difficult to interpret due to the absence of a control arm and small sample size.

Perampanel, a noncompetitive AMPAR antagonist, has shown cellular antitumor effects in preclinical studies and is effective as an antiepileptic in glioblastoma patients. It inhibits proliferation, cell invasion at the tumor infiltrative edge, and TM formation and dynamics [5]. The PerSurge trial investigates perampanel for recurrent or progressive brain tumors, aiming to inhibit tumor connectivity and growth [5,9]. Preliminary results of perioperative perampanel were safe and well tolerated, with peritumoral hyperexcitability similar to patients treated with levetiracetam, although maintenance therapy showed no impact on survival [31]. The limitation of pan-AMPA receptor inhibitors is that they may narrow the therapeutic window, suggesting the need to explore more specific inhibitors of the neuron–glioma synapse [5].

Riluzole, used in the treatment of amyotrophic lateral sclerosis [83], modulates glutamate release [8]. Its third-generation prodrug, troriluzole, reduces synaptic glutamate by enhancing astrocyte reuptake and inhibiting release by modulating sodium and calcium channels [8]. Riluzole also promotes apoptosis in glioma cells by antagonizing mGlu3 [2,42]. These multiple mechanisms of action may limit glioma progression and improve outcomes. Troriluzole is being evaluated in the GBM AGILE trial for newly diagnosed and recurrent glioblastoma [8].

Gabapentin and pregabalin are drugs that can modulate synaptogenesis by binding to the α2δ-1 subunit of voltage-sensitive calcium channels (VSCCs), a promising target in the context of cancer neurology [5,84]. Gabapentin is also included in the GLUGLIO trial [8,25].

### 5.2. GABAergic Neurotransmission

GABAergic signaling in the context of gliomas is a highly complex field of research, which differs significantly from its conventional role as an inhibitory neurotransmitter in the healthy adult brain. This particularity is primarily due to alterations in chloride homeostasis within tumor cells and their microenvironment (Table 1). In mature neurons of the central nervous system, the intracellular concentration of chloride is normally kept low by the K^+^-Cl^−^ cotransporter (KCC2), leading to hyperpolarization (chloride influx) when GABAergic A-type receptors (GABA_A_Rs) are activated, mediating inhibition.

However, in glioma cells, especially in DMG, overexpression of the Na^+^-K^+^-2Cl^−^ cotransporter (NKCC1), which brings chloride into the cell, is observed. In parallel, KCC2 expression is significantly reduced or even suppressed in these tumor cells and peritumoral tissue. This imbalance between NKCC1 and KCC2 results in an elevated intracellular chloride concentration in glioma cells. Under these conditions of high intracellular chloride concentration, activation of GABA_A_Rs receptors in glioma cells induces an outflow of chloride ions, which in turn causes depolarization of the cell membrane. This depolarizing effect transforms GABAergic signaling from inhibitory to excitatory in malignant cells. Membrane depolarization of glioma cells is sufficient to drive their proliferation and tumor growth. In fact, GABAergic interneuron activity has been shown to promote DMG proliferation in vivo [12,19], modulate the excitability of local neuronal circuits [1,3] and alter cortical processing through this induced hyperexcitability at the circuit level [1,62].

Neuron-to-glioma GABAergic synapses have been observed not only in DMGs. The application of GABA (1 mM) to small cell lung carcinoma (SCLC) cells in hippocampal allografts also caused an increase in GCaMP6s fluorescence, which indicates a calcium influx responsible for the excitatory response in these cancer cells [28]. This GABAergic excitation contributes not only to the neuronal network hyperexcitability but also to glioma-associated epilepsy [2,4,19,26,85].

The excitatory nature of GABAergic signaling appears to vary between glioma subtypes. Thus, while DMGs exhibit significant depolarizing GABAergic currents, hemispheric high-grade gliomas (HDI-WTs) may show minimal currents. This difference highlights the intrinsic heterogeneity of GABAergic biology among different types of gliomas.

In terms of therapeutic implications of GABAergic neurotransmission, administration of benzodiazepines, such as lorazepam, which enhance GABAergic signaling, has been shown to increase glioma proliferation and growth and shorten survival in patient-derived xenograft models of DMG [10,19]. GABA receptor inhibitors such as flumazenil and gabazine are being evaluated as therapeutic candidates [26]. This underscores the importance of considering the protumorigenic effects of certain drugs acting on the GABAergic system that clearly influenced excitotoxicity and seizures in gliomas [64]. In contrast, pharmacological inhibition of NKCC1 with bumetanide may counteract the depolarizing effect of GABA by shifting the GABA reversal potential (EGABA) to more negative values, suggesting a possible pathway to reduce tumor growth.

Suppression of GABA_A_ receptor subunit GABRG2 expression in glioma cell lines has been achieved by cloning and generation of glioma reporter cells [19]. Studies with GL261-GFP, a murine glioma cell line, implanted in the mice brain demonstrated that deletion of the *Asic1a* gene (an acid-activated ion channel) in the brain reduces tumor growth and prolongs survival [6]. This is attributed to altered K^+^ channel activity and attenuation of AMPA receptor currents in glioma cells. Acid stimulation of neurons, which mimics the acidic environment of the tumor, can induce excitatory postsynaptic currents in *Asic1a*-dependent glioma cells. This suggests that the interaction between the acidic microenvironment and neuronal ion channels directly influences glioma excitatory activity, which could be a therapeutic target [6]. Finally, the interaction of glioma cells with neurons and mechanisms of neuronal plasticity are an active area of therapeutic research to disconnect brain cancer cells from the brain [10,22].

### 5.3. Other Neurotransmitter and Neuromodulatory Systems

Recent studies have expanded the understanding of neuron–glioma communication to include classic neuromodulatory circuits, which can significantly influence tumor progression (Table 1).

Cholinergic neuronal activity promotes the proliferation of DMGs in a circuit-dependent manner. Midbrain cholinergic nuclei, such as the laterodorsal tegmentum nucleus (LDT) and the pedunculopontine nucleus (PPN), project to midline structures where DMGs arise, highlighting their potential to modulate DMG pathophysiology. Optogenetic stimulation of cholinergic neurons in the LDT increased proliferation in thalamic allografts, while stimulation of the PPN promoted proliferation in pontine allografts. This effect mirrors the circuit-dependent proliferative response observed in healthy oligodendrocyte precursor cells (OPCs) [13].

The proliferative effect of acetylcholine (ACh) is primarily mediated by muscarinic signaling through the M1 (CHRM1) and M3 (CHRM3) receptors, which are highly expressed in the OPC-like malignant cellular subpopulation of DMG. Pharmacological or genetic blockade of both M1 and M3 receptors was necessary to abolish the cholinergic activity-driven DMG proliferation in vitro and in vivo. ACh also enhances the migration of DMG cells in a dose-dependent manner. Furthermore, evidence suggests a bidirectional interaction: DMG cells reciprocally increase the activity of midbrain cholinergic neurons, progressively elevating midbrain cholinergic neuronal activity and ACh release over the course of the disease [13].

In addition to direct acetylcholine signaling, cholinergic neuronal activity influences DMG cells through the release of the paracrine factor BDNF. Specifically, explants of cholinergic midbrain nuclei exhibited activity-regulated BDNF release that promoted DMG proliferation, although the main mechanism driving growth in the pons and thalamus was found to be acetylcholine acting on M1 and M3 receptors [13].

Dopamine receptor signaling is also implicated in various cancers, including GBM, and is a recognized therapeutic target. Glioblastoma stem-like cells (GSCs) utilize the dopamine receptor D2 (DRD2) signaling pathway to promote their survival and proliferation. Dopamine receptor agonists, such as 7-OH-DPAT and quinpirole, increase the short-term proliferation and clonogenic growth of patient-derived GBM cells, an effect that is abrogated by DRD2 knockdown [86].

The oncogenic mechanism of DRD2 signaling involves interaction with the MET receptor tyrosine kinase. Agonist treatment significantly and rapidly increases the physical interaction between DRD2 and MET proteins, leading to the activation and phosphorylation of MET and its downstream effectors like GAB1, which drives GBM clonogenic growth and stemness. Conversely, pharmacological inhibition of DRD2 (e.g., with perphenazine, ONC201, or ONC206) inactivates the MET and STAT3 pathways [86].

Furthermore, DRD2 inhibition induces GBM cell death through a distinct mechanism: it promotes the acute interaction of DRD2 with the TRAIL receptors (death receptors 4 and 5). This interaction triggers the formation of a death-inducing signaling complex (DISC) in a TRAIL ligand-independent manner, leading to apoptosis. The inhibition of DRD2 with antagonists like perphenazine has been shown to extend the survival of GBM tumor-bearing mice [86].

A subset of GBMs (approximately 15% of specimens) harbors a tumor-autonomous dopamine-DRD2 signaling axis, characterized by the expression of dopamine biosynthesis enzymes, such as tyrosine hydroxylase (TH), and high levels of secreted dopamine. High TH mRNA levels correlate with worse prognosis in GBM patients. The DRD2-MET signaling axis is particularly activated in the mesenchymal GBM subtype, and high DRD2 levels portend poor survival for these patients [86].

Targeting these neuromodulatory pathways, such as the muscarinic M1/M3 signaling pathway in DMG and the DRD2 receptor in GBM, represents a promising novel therapeutic avenue for highly aggressive tumors [86].

### 5.4. Secreted Factors and Paracrine Signaling

Communication between neurons and glioma cells through secreted factors and paracrine signaling also reveals complex mechanisms that drive tumor progression (Table 2). Neuroligin-3 (NLGN3) has been identified as a highly important secreted factor in neuron–glioma interaction [1,2,3,5,6,7,8,10,11,12,13,19,20,23,24,25,26,27,28,31,32,57,75,84]. This synaptic adhesion protein is released by neurons and also by oligodendrocyte precursor cells (OPCs) in response to neuronal activity [2,3,5,10,11,27,28,31,32]. NLGN3 secretion is indeed a requirement for high-grade glioma progression in xenograft models. It functions as a potent mitogen, promoting the proliferation and growth of glioma cells [10,27,32,76]. Secreted NLGN3 binds to receptors on tumor cells, triggering activation of various oncogenic signaling pathways, including PI3K-mTOR, SRC, and RAS pathways [2,3,5,26,32]. The PI3K-Akt-mTOR pathway, in particular, is essential for NLGN3-induced signaling and glioma growth (Figure 1) [32,50]. Furthermore, studies indicate that NLGN3 not only promotes glioma cell proliferation but also induces a positive feedback loop, where tumor cells themselves increase NLGN3 expression and secretion, amplifying oncogenic signaling and creating a self-sustaining stimulation of tumor growth [2,3,5,10,11,27,28,31,32]. NLGN3 expression inversely correlates with overall survival of glioma patients [2,32].

Another crucial mediator in neuron–glioma communication is brain-derived neurotrophic factor (BDNF). Its secretion is regulated by neuronal activity, and its release into the tumor microenvironment activates the BDNF/TrkB (NTRK2) pathway in glioma cells [5,29,30]. This pathway not only promotes glioma cell proliferation but also increases the amplitude of glutamate-induced excitatory postsynaptic currents (EPSCs) and facilitates AMPA receptor trafficking to the glioma cell membrane through CAMKII-dependent calcium signaling, thereby modulating synaptic strength [29,30]. Thus, BDNF-TrkB signaling potentiates synaptic integration of tumor cells into neuronal networks, promoting neuron–glioma synapse formation and glioma plasticity [10]. Genetic or pharmacological inhibition of TrkB has been shown to be effective in robustly inhibiting tumor progression and prolonging survival in xenograft models [29,30,79].

Other synaptogenic factors secreted by glioma cells include thrombospondin-1 (TSP-1) and SPARCL1. TSP-1, a protein secreted by glioblastoma cells, promotes the formation of new synapses and remodeling of neuronal circuits [4,10,11,22,26,84]. In particular, TSP-1 binds to the α2δ-1 receptor on neurons, a component of voltage-sensitive calcium channels, which promotes synaptogenesis and contributes to neuronal hyperexcitability [5,10,22,25,84]. TSP-1 is also implicated in TMs formation [22]. SPARCL1 (hevin), highly expressed in glioma cells, especially in astrocytic tumors, is also associated with synaptogenesis [23]. It is found in the synaptic enrichment area of the brain and in glial cell prolongations surrounding synapses [23,84]. Overexpression of SPARCL1 significantly increases neuron–glioma synapse formation in TMs [23]. This synaptogenic activity of TSP-1 and SPARCL1 creates a microenvironment that not only favors tumor growth but also the formation of aberrant neuronal circuits and neuronal hyperexcitability, which may have a negative impact on cognitive function and patient survival [11,22,62].

Insulin-like growth factor 1 (IGF-1) has been identified as another significant mediator in the complex interaction between neurons and gliomas. Its release by specific neurons, such as mitral and tufted cells of the olfactory bulb, occurs in response to sensory activity. This sensory experience-dependent release establishes a mechanism by which physiological neuronal activity can inadvertently promote tumor growth and gliomagenesis. For example, olfactory sensory experience has been shown to promote gliomagenesis, and this is attributed to neuronal activity-dependent paracrine secretion of IGF-1 in such cells [5,26,27,37]. This factor also promotes tumor cell proliferation and tumor growth [5,26,27,37]. Studies suggest that IGF-1 secretion and synaptic pathways might be independent, based on in vitro co-culture experiments [5]. However, it has been suggested that it would be relevant to understand how IGF-1 secretion could modulate neuron–glioma synapses in other model systems involving a neural microenvironment [5]. Importantly, IGF-1 is one of many factors that can be overexpressed by neuronal hyperexcitability. In addition, pharmacological inhibition of IGF-1R has shown promising antitumor effects, inducing apoptosis, inhibiting growth, and reducing tumor size [5].

LGI1 (Leucine-rich glioma inactivated 1) regulates synapse number and synaptic activity in neurons of the hippocampus and cortex [87,88,89,90,91,92,93,94]. LGI1 autoantibodies increase the probability of synaptic release by reducing the density of Kv1.1 and Kv1.2 channels, leading to a widening of the presynaptic action potential [60,91,92,93]. LGI1 interacts with A disintegrin and metalloproteinase 22 (ADAM22), and its activity is proposed to suppress NgR1-mediated synaptic repression or deletion [60,91,92,93]. The actual role of LGI1 in brain tumor progression is unclear, and studies have been contradictory and scarce. Whereas some studies have shown that LGI1 is downregulated in glioblastoma cell lines and tumors, suggesting a possible function as a tumor suppressor, other studies have indicated that LGI1 mRNA expression is present in approximately 50% of glioblastoma tumors and have suggested that LGI1 controls the proliferation and invasiveness of glioma cell lines, implying a more likely role in tumor progression toward malignancy [95]. However, this latter function has been questioned. In fact, a previous study using a non-specific antibody did not detect LGI1 signals in tumor tissues, interpreting this as supporting a tumor suppression function, but these results were considered unreliable due to the non-specificity of the antibody. A current study found detectable levels of the LGI1 protein in approximately one-third (8 of 24, 33%) of high-grade glioma tumors (glioblastoma or anaplastic astrocytoma). LGI1 levels were generally lower than in normal cortical tissues, except in two samples [95].

Other factors include fibroblast growth factors (FGFs), secreted glycoproteins that induce synaptic vesicle accumulation and control neuronal development [84], as well as semaphorins (Sema3s), which act as positive and negative retrograde synaptic organizers. Furthermore, overexpression of Sema4F has been found to lead to increased excitatory and decreased inhibitory synapses in mouse tumors [58,59].

### 5.5. Ion Channels and Membrane Potential Regulation

Interaction between glioma cells and surrounding neurons through ion channels and membrane potential regulation is a critical aspect in tumor progression (Table 3). Acid-sensitive ion channel 1a (ASIC1a) plays a distinctive role in this communication [6]. The tumor microenvironment is characterized by marked acidification, mainly due to the Warburg effect, where cancer cells metabolize glucose predominantly through glycolysis, even in the presence of oxygen, resulting in the accumulation of lactic acid [43]. This acidic environment with pH levels that can range from 7.4 to 6.2 units and even drop to 5.5–3.4 units or lower influences surrounding neurons. Acidification activates ASIC1a channels in surrounding neurons, which are proton receptor dominant. Activation of ASICs in neurons leads to the influx of Na^+^ and/or Ca^2+^ ions, resulting in the release of neurotransmitters and the initiation of signaling pathways [6]. Preliminary studies revealed that local application of acid to neurons in high-grade glioma brain slices induces postsynaptic currents in glioma cells. Activation of ASIC1a in neurons can also modulate synaptic neurotransmitter release in response to extracellular acidification [6].

Genetic studies have shown that deletion of ASIC1a in mice with implanted glioma leads to a significant reduction in tumor size and increased survival [6]. Specifically, deletion of ASIC1a in excitatory neurons (Asic1aVglut1-cKO) also reduces tumor size, underscoring the substantial impact of brain ASIC1a on high-grade glioma progression, primarily through interactions between excitatory neurons and glioma cells. In addition, pharmacological inhibition of neuronal ASIC1a has also shown potential to inhibit high-grade glioma progression [6]. These findings suggest that tumor acidification is not simply a metabolic by-product but an active mechanism by which glioma cells can modify the surrounding neuronal activity for their own benefit. Brain ASIC1a expression and activity are elevated after glioma implantation, which could represent an attempt by neurons to counteract the inhibitory effects of the acidic environment on neuronal signaling [6].

Potassium channels also contribute to neuron–glioma communication and tumor pathophysiology [2,6]. Alterations in potassium currents in glioma cells affect their excitability and responsiveness to synaptic signals. Potassium channels have been observed to regulate a wide range of biological processes and are crucial in various diseases, including their selective expression in numerous tumor cells, where they impact biological processes such as proliferation, apoptosis, differentiation, and invasion [6]. For example, a subpopulation of highly connected, “pacemaker-like” glioma cells exhibits rhythmic Ca^2+^ oscillations that are dependent on the calcium-activated potassium channel KCa3.1 [3,11]. Blockade of KCa3.1 suppresses these oscillations of the glioma cell autonomous network and prolongs survival in animal models. In human patients, high tumor expression of KCa3.1 is associated with reduced survival [3,11]. In addition, decreased K^+^ current has been observed in glioma cells implanted in ASIC1a-deleted brains, suggesting a possible influence of ASIC1a on potassium channel functions in glioma [6]. Distinct K^+^ current profiles, notably the intermediate rectifier K^+^ current, are unique features of the implanted glioma mouse model, and their reduction in the absence of ASIC1a suggests a role in neuron–glioma communication. Pharmacological inhibition of voltage-activated K^+^ channels has been shown to reduce cancer cell proliferation [6].

Furthermore, ionotropic purinergic receptors such as the P2X7 receptor (P2X7R), activated by extracellular ATP binding, are functionally expressed in glioma cells and contribute to malignant characteristics, including intracellular Ca^2+^ mobilization. Activation of P2X7R in C6 glioma cells has been shown to increase proliferation and cell mobility. Notably, the P2X7R acts synergistically with Ca^2+^-permeable AMPA receptors to further increase Ca^2+^ influx in tumor cells. While activation of P2X7R may induce cell death in radiosensitive glioma cells, its suppression can sometimes promote glioma growth through the upregulation of growth factors like EGFR [96].

## 6. Epigenetic and Transcriptional Regulation

Synaptic interaction between neurons and glioma cells not only triggers immediate signaling cascades but also results in profound changes in gene expression that, in turn, fundamentally reshape the phenotype of tumor cells [20]. This process is an emerging focus in the field of cancer neurophysiology, which seeks to understand and therapeutically target these complex bidirectional interactions between the nervous system and disease.

A crucial aspect of this interaction at the genetic level is the remodeling of the promoter–potentiator interactome in glioblastoma [20]. This reorganization involves profound modification of promoter–promoter interactions, chromatin accessibility, and redistribution of histone marks within the glioblastoma. As a result of these changes in the regulatory and topological landscape, a significant loss of long-range regulatory interactions and general promoter activation is observed, which orchestrates specific alterations in gene expression. These affected genes are associated with diverse processes, including glutamatergic synapses, axonal guidance, axonogenesis, and chromatin binding/remodeling [20].

Importantly, such modifications in gene expression are consistent across the four glioblastoma subtypes analyzed [20]. Furthermore, glutamate receptor signaling shows a stronger association with glioblastoma samples compared to lower-grade gliomas. Within this framework, the transcription factors SMAD3 and PITX1 have been identified as key direct regulators of a set of target genes involved in synaptic contacts and axonal guidance [20]. Indeed, inhibition of SMAD3 and stimulation of neuronal activity have been shown to cooperate additively to promote glioblastoma cell proliferation [20].

In the field of epigenetic regulators, chromodomain DNA-binding helicase 2 (CHD2) exerts a particularly important role [49]. CHD2 is critical for the control of the epigenome and the expression of axonal and synaptic guidance genes in DMGs, which in turn promotes neuronal-induced proliferation and pathogenesis of these highly lethal tumors. Research has revealed that deletion of CHD2 in MGD cells of the H3.1K27M subtype compromises their cell viability, impairs neuron–glioma synaptic connections in vitro, decreases neuron-induced proliferation both in vitro and in vivo, reduces activity-dependent calcium transients in vivo, and prolongs survival of DMG H3.1K27M-bearing mice [14,49,97].

All this suggests that CHD2 regulates specific genetic programs linked to synaptic connectivity. CHD2 exerts its control by regulating histone modifications, including the levels of H3K27me3, H3K27ac, and H3K4me3 at the promoters of these genes. In addition, CHD2 co-localizes and up-regulates gene expression together with the transcription factor FOSL1, establishing a FOSL1-CHD2 axis that controls axonal and synaptic guidance genes, which promotes neuron–glioma interaction and tumor progression, specifically in the H3.1K27M subtype of DMG [14,49].

It is plausible that different glioma subtypes employ distinct mechanisms to modulate their interaction with neurons, given that CHD2 dependence has been observed particularly in H3.1K27M DMG cells and not in H3.3K27M cells.

## 7. Mechanisms of Facilitation of Tumor Invasion

Glioma cells have developed a remarkable ability to “hijack” normal neuronal mechanisms to facilitate their invasive dissemination through brain tissue [26]. This integration of tumor cells into the brain parenchyma not only alters brain functionality in patients but also accelerates cancer progression [1,10]. To facilitate tumor invasion, glioma cells have developed several mechanisms.

### 7.1. Neuronal-like Phenotype and Non-Connected Cells

Non-connected tumor cells are predominantly found in the tumor periphery and are the drivers of glioma invasion [5]. These cells exhibit high invasiveness and express genes characteristic of a neuronal or oligodendroglial progenitor-like (NPC/OPC-like) cell state, with neuronal pathway-based signaling and neurodevelopmental signatures [5]. This adoption of a “neuronal-like” or “neural-progenitor-like” phenotype is crucial for their migratory behavior, and it has been suggested that gliomas adopt neuronal properties for invasion [11]. Indeed, remodeling of human neuronal circuits by glioblastoma decreases survival [22]. Glioma plasticity may even recapitulate neuronal plasticity established during memory formation in the adult brain [10].

### 7.2. The Central Role of Tumor Microtubules (TMs)

Tumor microtubules (TMs) are ultra-long membranous extensions that glioma cells use to scan the brain, invade, and form a therapy-resistant network. TMs are the predominant location of neuronal interaction [5]. The formation, elongation, and dynamics of TMs are directly influenced by synaptic signaling [3,5,21]. Activation of AMPA receptors, especially located on TMs, drives their formation, whereas inhibition of AMPA receptor activity, for example with perampanel, reduces the formation of TMs [9]. Synaptic signaling also induces intracellular calcium waves that propagate through the glioma network connected by TMs, promoting invasion and proliferation [26].

In addition, glioma cells with TMs have demonstrated enhanced tropism for mature neurons [2]. TMs share morphological and molecular similarities with axonal growth cones and neurites during neurodevelopment [5,8]. Genes such as GAP43 and TTYH1 are important drivers of TM extension and cell invasion [3,5]. CHD2 depletion in H3.1K27M MGD cells significantly reduced the proportion of DGM cells with TMs, as well as their length, when co-cultured with neurons, suggesting a role in neuron–glioma interaction [14,49].

### 7.3. Induction of Matrix Metalloproteinases (MMPs)

Synaptic activity contributes to tumor invasion also through induction of MMPs. Glioblastoma cells “vampire” WNTs from neurons and trigger a JNK/MMP signaling loop that enhances glioblastoma progression and neurodegeneration [5,24]. Glioma cells release soluble factors that induce the expression of MMPs in tumor-associated microglia cells (TAMs), such as MMP-2 and MT1-MMP, which in turn promote tumor invasion [11,48]. Interleukin-6 (IL-6) secreted by human astrocytes also upregulates MMP14 expression in glioma cells, which plays a critical role in promoting their migration and invasion [10]. In addition, perineuronal networks, which act as electrostatic insulators, are degraded in a distance-dependent manner by glioma-released MMPs, amplifying the loss of GABAergic inhibition and glutamate-induced neuronal death [3].

### 7.4. Axonal Projections as Migration Pathways

Glioma cells infiltrate the brain generally along organized anatomical structures such as blood vessels and white matter tracts, which contain neuronal axons. This suggests the involvement of neuronal populations [7]. Infiltrating tumor cells at the invasion front are enriched in axonal guidance genes [7,49]. A key regulator in this process is Sema4F, which has been identified as a key mediator of activity-dependent infiltration and promotes synaptic remodeling and brain hyperactivity [3,7,10]. CHD2 depletion in H3.1K27M MGD cells, a chromatin remodeler, affects the expression of axonal guidance and synaptic genes, such as EPHB3, which has known roles in axonal guidance and excitatory synapse formation, which in turn compromises neuron–glioma interaction and neuron-induced proliferation [49]. In addition, synapses facilitate tumor cell dissemination by allowing them to migrate along neuronal structures [26].

### 7.5. Modification of Biomechanical Properties

Synaptic signaling modifies the mechanical properties of glioma cells by making them more deformable. The cytoskeletal composition of TMs includes F-actin and microtubules [5], with F-actin polymerization being essential for TM formation and glioma invasiveness [2]. Evidence also suggests that neuronal activity drives the generation of infiltrating glioma populations and that these populations correspond to the invasion front of tumors [7]. Studies have identified that proteins involved in filopodia formation, such as Arp3 (an important modulator of the neuronal cytoskeleton), are present in exosomes derived from more aggressive gliomas and are associated with modification of synaptic efficiency and formation of new neuronal branches [18]. This suggests that changes in the actin cytoskeleton are central to the ability of glioma cells to interact with their microenvironment and migrate. Glioblastoma invasion may also involve epithelial-mesenchymal transition (EMT), a process that alters cell morphology and motility, driven by secreted factors such as TGF-β, fibroblast growth factors (FGFs), and epidermal growth factor (EGF) [10,98].

## 8. Resistance to Therapies

Glioma cells have evolved complex mechanisms to resist standard therapies, which is largely attributed to the formation of TMs and their synaptic integration with neurons [10]. The interconnected network created by glioma cells allows their resistance to surgery, radiotherapy, and chemotherapy [29,30]. Highly connected glioma cells survive treatment and can form more TMs to interconnect more densely, which facilitates self-repair. Even after targeted destruction of key “pacemaker cells” in the network, the network can adapt by recruiting new cells to restore lost functionality [5,29]. In contrast, tumor cells that are not connected are more sensitive to treatment and tend to die [4]. Genes such as GAP43, TTYH1, and CHI3L1 play a crucial role in the formation and function of TMs, promoting network growth and resistance [10].

In addition to microtubule networks, the functional and “genuine” electrochemical synapses created by glioma cells with neurons, acting as the postsynaptic partner, are also responsible for glioma resistance [10]. These synapses are predominantly found in the glioma infiltration zone [71,72,73] and are mainly mediated by glutamatergic AMPARs [5,29,30]. Furthermore, the involvement of NMDA receptors in brain metastases and possibly gliomas has also been reported [26]. Neurons transmit growth and invasion signals to the tumor through these synapses [27,29,30]. Synaptic input, particularly through AMPARs, induces calcium transients in glioma cells, which propagate through the network of TMs and promote invasion and proliferation [5,26,29,30]. Indeed, blockade of Ca^2+^-permeable AMPARs suppresses migration and induces apoptosis in glioma cells [5,26]. Increased trafficking of AMPARs to the glioma cell membrane, promoted by BDNF-TrkB signaling, leads to an increase in the amplitude of glutamate-evoked currents, and increased depolarizing current amplitude promotes increased glioma proliferation. These mechanisms contribute to the tumor’s ability to resist therapy [29,30].

Synaptic signaling can also activate specific drug resistance pathways in glioma cells. Activation of NMDARs in human glioblastoma cells promotes cell survival and migration [26]. Furthermore, antagonizing NMDARs increases radiosensitivity by impairing the ability to repair DNA double-strand breaks, suggesting a mechanism by which synaptic signaling may support the restoration of radiation-induced damage [2,26]. It has also been hypothesized that activation of AMPA receptors may induce the expression of efflux transporters that remove chemotherapeutic agents from tumor cells [5,29,30]. Therefore, disruption of these malignant networks is presented as a promising therapeutic strategy to improve treatment efficacy [5,10,22] (Table 4).

## 9. Open Questions and Controversies in Neuron–Glioma Synapse Research

While the discovery of neuron–glioma synapses has fundamentally shifted the understanding of brain cancer, several key controversies, context dependencies, and methodological limitations currently occur [30].

### 9.1. Context-Dependent Signaling and Tumor Heterogeneity

The functional role of several key signaling pathways is highly dependent on the glioma subtype, reflecting the cellular and genetic heterogeneity inherent in these tumors [2].

The role of GABA signaling is context dependent. While GABA typically acts as an inhibitory neurotransmitter in the mature healthy adult brain, its activation in certain gliomas, notably Diffuse Midline Gliomas (DMGs), is depolarizing and growth-promoting. This reversal of function is attributed to the overexpression of the NKCC1 cotransporter and reduced KCC2 expression, leading to high intracellular chloride concentrations. However, this excitatory nature appears to vary between glioma subtypes, as hemispheric high-grade gliomas (HDI-WTs) may exhibit only minimal GABAergic currents. This complexity necessitates careful consideration when designing therapies that modulate the GABAergic system, as benzodiazepines, which enhance GABAergic signaling, have been shown to increase DMG proliferation and shorten survival [85].

The precise function of leucine-rich glioma inactivated 1 (LGI1) in brain tumor progression remains ambiguous, with contradictory reports in the literature. LGI1 regulates synapse number and synaptic activity in healthy neurons. Some evidence suggests LGI1 acts as a tumor suppressor, noting its downregulation in glioblastoma cell lines and tumors. Conversely, other studies have detected LGI1 mRNA expression in approximately 50% of glioblastoma tumors and suggested a role in promoting proliferation and invasiveness, implying a pro-malignant function. Although LGI1 protein is generally found at lower levels in high-grade gliomas compared to normal cortex, its role is not uniformly suppressed, reinforcing the need to clarify its context-specific function [2].

Even within the highly aggressive DMG category, mechanistic heterogeneity exists. The dependence on the epigenetic regulator CHD2 (chromodomain DNA-binding helicase 2) and its control over axonal and synaptic guidance genes has been observed particularly in the H3.1K27M DMG subtype, but not in H3.3K27M cells. This suggests that different glioma subtypes employ distinct mechanisms to modulate their interaction with neurons, complicating universal therapeutic targeting [30].

### 9.2. Unresolved Synaptic Characteristics

The electrophysiological evidence confirming neuron–glioma synaptic functionality relies heavily on the demonstration of evoked excitatory postsynaptic currents (EPSCs) in response to neuronal stimulation. However, the absence of classical miniature postsynaptic currents (mEPSCs), which represent spontaneous neurotransmitter release events observed in conventional functional synapses, is lacking. Much of the defining functional evidence relies on recordings that demonstrate evoked responses, where stimulation is applied directly to neurons (e.g., optogenetically or electrically). While the detection of mEPSCs in glioma cells is necessary to confirm a functional, autonomously active synapse, obtaining stable, high signal-to-noise ratio recordings needed to capture miniature events in vivo is technically highly demanding, even in normal healthy neurons [30,72,74]. Furthermore, the malignant AMPARs expressed on glioma cells may not be continuously activated in monoculture (or in certain preparation contexts), and some in vitro studies found that specific co-culture or in vivo conditions were necessary to observe the oncolytic effects of AMPAR antagonists. This suggests that the spontaneous firing necessary to trigger measurable mEPSCs may be suppressed or simply rare outside of an active circuit environment. While fast EPSCs lasting a few milliseconds have been reported in glioma cells, mirroring AMPAR kinetics, studies reporting synaptic functionality often focus on the larger evoked EPSCs or prolonged slow inward currents (SICs), whose origins can be partially non-synaptic (e.g., mediated by potassium accumulation or glutamate transporters). This differentiation suggests the complexity of the signal environment [6,29,30,72,74].

Finally, the absence or rarity of mEPSCs may reflect a true biological difference between the malignant neuron–glioma synapse and canonical neuronal synapses, where the machinery governing neurotransmitter release probability might be aberrantly regulated [6,30,74].

### 9.3. Methodological Limitations and Translational Challenges

The clinical translation of findings from neuron–glioma research is hindered by inherent methodological challenges and the difficulty of accurately translating preclinical data to human outcomes. While studies in animal models and human tissue confirm the existence of electrochemical synapses, the full complexity of human cognitive alterations remains difficult to model accurately. For instance, ECoG studies in human patients show fundamental modifications in cortical processing, such as decreased entropy of temporal signals and diffuse network recruitment during cognitive tasks, which reflects a loss of computational complexity. These subtle alterations, which contribute to cognitive deficits and poor quality of life, are challenging to fully capture in preclinical models [30].

Therapeutic strategies, such as using pan-inhibitors of AMPA receptors (e.g., Perampanel), may be limited in their therapeutic window due to the receptors’ critical roles in normal neuronal function. The challenge lies in developing more specific inhibitors of the malignant neuron–glioma synapse to avoid significant neurological side effects [30].

While the link between synaptic activation and therapeutic resistance is clear, the exact mechanisms for resistance are still being elucidated. For example, it is hypothesized that the activation of AMPA receptors may induce the expression of efflux transporters that remove chemotherapeutic agents from tumor cells, but this remains a hypothesis requiring verification [30].

Addressing these open questions, particularly concerning tumor heterogeneity, context-dependent roles of neurotransmitters, and the precise molecular mechanisms of resistance, is essential for designing successful precision medicine approaches aimed at disconnecting cancer cells from the neural circuits [26].

### 9.4. Barriers of Therapeutic Translation

While preclinical studies provide strong validation that targeting neuron–glioma synapses is a promising strategy to disconnect brain cancer from neural circuits, the clinical translation of these approaches requires a balanced and critical evaluation. The optimism surrounding novel targets must be contextualized by the significant barriers inherent to neuro-oncology trials, including tumor heterogeneity, specificity concerns, and early clinical limitations [30].

#### 9.4.1. Limitations of Clinical Efficacy and Trial Design

The clinical data available for repurposed drugs targeting neuroglial signaling are still preliminary and limited. Trials such as the phase Ib/II GLUGLIO trial, which combines glutamate signaling inhibitors (memantine, gabapentin, and sulfasalazine) with standard chemoradiotherapy, have faced difficulties in interpreting efficacy results due to the absence of a control arm and small sample size. Despite strong preclinical rationale, some targeted interventions have not met efficacy endpoints. For instance, a small phase I study of sulfasalazine monotherapy (an xCT inhibitor aiming to reduce glutamate excitotoxicity) in advanced glioblastoma was terminated for lack of efficacy. Early results from the PerSurge trial (NOA-30), which investigates the AMPA receptor antagonist Perampanel around surgery, suggest that while the drug is safe and well tolerated perioperatively, maintenance therapy showed no impact on survival. This suggests that the timing, dose, and combination strategy may be crucial, and that monotherapy may be insufficient to overcome therapeutic resistance conferred by the robust tumor network [2].

#### 9.4.2. Specificity Concerns and Adverse Effects

A major barrier to clinical adoption is the difficulty in selectively targeting the malignant neuron–glioma synapse without interfering with normal synaptic transmission, which is essential for healthy brain function. Inhibitors of pan-receptors, such as pan-AMPA receptor antagonists like Perampanel, may be limited by a narrow therapeutic window. Since AMPA receptors are critical for glutamatergic neurotransmission throughout the CNS, systemic inhibition carries a risk of significant off-target effects on normal synapses and neurological function. This highlights the need to develop more specific inhibitors of the malignant neuron–glioma synapse to avoid severe neurological side effects [29,30,56,63].

Certain modulators of neuronal activity can be counterproductively protumorigenic, illustrating the risk of general neuromodulation. For example, benzodiazepines, which enhance inhibitory GABAergic signaling, have been shown to increase glioma proliferation and growth and shorten survival in DMG models. This is due to the paradoxical, depolarizing nature of GABA signaling in these specific tumor cells, emphasizing that the therapeutic strategy must be tailored to the context-dependent mechanisms of the tumor subtype [19,26,30].

#### 9.4.3. Impact of Tumor Heterogeneity

Gliomas are highly heterogeneous tumors, characterized by multiple cell subpopulations (including GSCs) and significant genetic and phenotypic diversity. The functional integration of gliomas into neural circuits still needs precise patient stratification. Since synaptic mechanisms (such as the depolarizing role of GABA or the dependence on specific epigenetic regulators like CHD2) vary between tumor subtypes (e.g., DMG vs. high-grade hemispheric gliomas), a “one-size-fits-all” approach to antineuronal therapy is likely to fail. Successful translation requires identifying the specific neuro-malignant axis activated in each patient’s tumor [5,30].

Clinical studies have shown that high functional connectivity (HFC) within glioblastoma is a significant predictor of significantly lower overall survival. While HFC identifies the most aggressive tumors, it also suggests that these highly interconnected networks are robust and potentially require multimodal therapies (combination therapies) rather than single-target approaches [29,30,71,72].

## 10. Impact on Survival

Clinical studies have established a direct correlation between functional connectivity within glioblastoma and patient survival [3,22]. Patients whose tumors exhibit high functional connectivity (HFC) have significantly lower overall survival compared to those with low connectivity [22]. A survival analysis in humans revealed that glioblastoma patients with intrinsic functional connectivity had an overall survival of 71 weeks, while those without HFC had a survival of 123 weeks, demonstrating a striking inverse relationship [22]. This correlation is relevant even after controlling for other important prognostic variables such as age, tumor volume, treatment completion, and extent of tumor resection. This functional integration of gliomas into neural circuits, in addition to impacting survival, also negatively influences cognition [1,3], which is observed in language task performance. The glioma-infiltrated cortex has a reduced capacity for information encoding, and temporal decoding strategies were not feasible [1].

HFC is associated with several markers of tumor aggressiveness. Glioma cells with HFC exhibit a fivefold increase in proliferation when cultured with neurons, unlike low-connectivity cells that do not show such an increase. HFC regions also show an increased expression of the proliferative marker Ki-67 [22]. In addition, these HFC glioma cells show increased spheroidal invasion area and extend long prolongations as TMs in response to the neuronal conditioning medium. This increased synaptic activity, mediated by increased depolarizing current amplitude, promotes increased glioma proliferation [22,99]. In contrast, non-interconnected tumor cells are more sensitive to treatment and tend to die [29,30]. HFC regions are enriched with a glioblastoma subpopulation that exhibits a distinct synaptogenic and neurotrophic phenotype, producing factors such as TSP-1 that promote synapse formation and cell division [22].

Experimental studies in animal models have also confirmed that disruption of neuron–glioma communication can have significant therapeutic effects. Genetic deletion of the neuronal ASIC1a results in reduced tumor growth and significantly prolonged survival in glioma-bearing mice [6]. Mice without ASIC1a showed a substantial increase in overall survival and a reduction in tumor size, an effect that was maintained even when ASIC1a deletion was specific to excitatory neurons. ASIC1a deletion attenuates AMPA receptor-mediated excitatory postsynaptic currents in glioma cells and reduces NLGN3 expression [6].

In addition to genetic manipulation, several pharmacological interventions have demonstrated therapeutic potential by disrupting these malignant interactions. Gabapentin, by blocking the α2δ-1 thrombospondin receptor, reduces synaptogenesis, glioma proliferation, and network synchrony, improving survival in preclinical studies [22,26]. The noncompetitive AMPA receptor antagonist Perampanel has been shown to reduce proliferation, invasion, and TMs formation in preclinical studies [9,23,31,64]. Clinical trials such as PerSurge are investigating its efficacy in humans [9]. Genetic or pharmacological inhibition of the TrkB receptor (encoded by NTRK2) in glioma cells also inhibits tumor progression and substantially prolongs survival in xenograft models [29,30]. Inhibition of gap junctions with meclofenamate or bentamapimod can sensitize glioblastoma cells to chemotherapy and reduce tumor growth [5,8,10,26]. Memantine, a blocker of NMDA-type glutamate receptors, could prevent synapse formation between neurons and glioma cells, thereby decreasing tumor invasion and neuroglial signaling [8]. This drug is being investigated in a multicenter phase Ib/II clinical trial called GLUGLIO, in combination with gabapentin, sulfasalazine, and chemoradiotherapy [8]. It is proposed that next-generation precision medicine should attempt to switch off brain cancer cells to improve treatment outcomes [10]. Overall, these findings provide preclinical validation and support for the idea that targeting neuron–glioma interactions is a viable and promising therapeutic strategy to improve patient prognosis (Table 4).

## 11. Conclusions

Recent discoveries have challenged the traditional idea that gliomas are passive masses that cause damage solely by occupying space. On the contrary, glioma cells are active and adaptable elements of neural circuits, capable of forming authentic electrochemical synapses with neurons and mimicking neuronal activity to promote their own proliferation, invasion, and resistance to therapies. Synaptic communication between neurons and gliomas, particularly through glutamatergic signaling and, paradoxically, excitatory GABAergic signaling, appears to be fundamental in generating a hyperexcited tumor microenvironment that disrupts or alters nerve information processing. Molecular mediators such as neuroligin-3, BDNF, and IGF-1 reinforce a pro-tumorigenic feedback loop that integrates glioma cells into the network of brain connections. This integration not only drives tumor progression but also profoundly affects the cognition and survival of affected individuals. Highly connected gliomas exhibit synaptogenic characteristics and structural plasticity that facilitate brain invasion and therapeutic resistance, particularly through tumor microtubules and gap junction coupling. Therapeutic strategies targeting these neurogliomal circuits, including AMPA/NMDA receptor antagonists, chloride transporter inhibitors, synaptogenic protein blockers, and compounds that alter tumor microtubule dynamics, offer a promising avenue for disconnecting brain cancer from the brain itself. However, the clinical translation of these approaches still needs validation, precise patient stratification, and integration into multidisciplinary neuro-oncology protocols. Understanding brain cancer as a disease of pathological neuroplasticity, rather than simply as a proliferative malignant neoplasm, opens up a new avenue of research for the development of drugs that disrupt electrical and synaptic communication between neurons and glioma cells to improve treatments for this type of tumor.

## Figures and Tables

**Table 2:** Secreted factors driving glioma progression.

Factor	Source	Mechanism of Action/Receptor	Effect on Glioma
Neuroligin-3 (NLGN3) [29,30,32,50,71,72,73,79]	Neurons and oligodendrocyte precursor cells (OPCs).	Secreted in response to neuronal activity. Binds to receptors on tumor cells, activating oncogenic pathways (PI3K-mTOR, SRC, RAS).	Potent mitogen; promotes proliferation and growth. NLGN3 expression correlates inversely with overall survival.
Brain-derived neurotrophic factor (BDNF) [29,30,39,79]	Neurons.	Activates the BDNF/TrkB (NTRK2) pathway in glioma cells. Promotes AMPA receptor trafficking to the membrane.	Enhances proliferation, malignant synaptic plasticity, and increases the amplitude of glutamate-evoked currents.
Thrombospondin-1 (TSP-1) [5,22,29,30,79]	Glioblastoma cells.	Binds to the α2δ-1 receptor (VSCC component) on neurons.	Promotes the formation of new synapses, neuronal hyperexcitability, and TMs formation.
SPARCL1 (hevin) [23,26,29,30,79]	Glioma cells (especially astrocytic tumors).	Associated with synaptogenesis. Overexpression significantly increases neuron–glioma synapse formation on TMs.	Favors tumor growth and aberrant neuronal circuit formation.
Insulin-like growth factor 1 (IGF-1) [27,29,30,37,39,79]	Specific neurons (e.g., olfactory bulb cells).	Released in response to sensory/neuronal activity.	Promotes tumor cell proliferation and gliomagenesis.

**Table 3:** Ion channels and membrane potential regulation targets.

Target/Mechanism	Role in Glioma Interaction	Therapeutic Strategy	Clinical Status
AMPARs [29,30,63,79]	Mediate most excitatory postsynaptic currents (EPSCs); drive tumor microtubule (TM) formation and dynamics.	Perampanel (noncompetitive antagonist).	Reduces proliferation, invasion, and TMs formation. Under investigation for recurrent GBM (PerSurge trial).
xCT antiporter (SLC7A11) [5,29,30,79]	Responsible for massive non-synaptic glutamate release by glioma cells, causing excitotoxicity.	Sulfasalazine (inhibitor of xCT).	Reduces extracellular glutamate levels, decreasing tumor growth and alleviating excitotoxicity. Part of the GLUGLIO clinical trial.
NMDARs [6,29,30,79]	Activation promotes survival; antagonism increases radiosensitivity.	Memantine (noncompetitive antagonist).	May prevent neuron–glioma synapse formation. Being investigated in GLUGLIO trial.
NKCC1 cotransporter [30]	Overexpression in DMGs causes Cl^−^ accumulation, making GABA signaling depolarizing/excitatory.	Bumetanide (NKCC1 inhibitor).	Counteracts the depolarizing effect of GABA, suggesting a pathway to reduce tumor growth.
ASIC1a channel [6]	Activated in surrounding neurons by the acidic tumor microenvironment (TME). Activation induces neurotransmitter release.	Genetic deletion or pharmacological inhibition of neuronal ASIC1a.	Deletion reduces tumor size and significantly prolongs survival in animal models. Effective against high-grade glioma progression.
α2δ-1 subunit (VSCC component) [22,29,30,79]	Receptor for synaptogenic factor TSP-1. Also binds gabapentinoids.	Gabapentin/Pregabalin.	Reduces synaptogenesis and proliferation. Included in the GLUGLIO trial.
KCa3.1 channel [30]	Responsible for rhythmic Ca^2+^ oscillations in “pacemaker-like” glioma cells.	Pharmacological blockade.	Suppresses autonomous network oscillations and prolongs survival in animal models. High expression associated with reduced survival in patients.
P2X7R [30]	Ligand-gated cation channel activated by extracellular ATP. Contributes to Ca^2+^ mobilization.	Antagonism or agonism (context dependent).	Activation increases proliferation/mobility and acts synergistically with AMPAR to increase Ca^2+^ influx. Inhibition can sometimes promote growth by upregulating EGFR.
T-type VGCCs (Cav3.1 channels) [30]	Ca^2+^ channels involved in proliferation and cell survival pathways (mTOR/Akt) in GBM stem-like cells.	Pharmacological blockade (e.g., Mibefradil).	Inhibition reduces cell survival/proliferation and induces apoptosis. Mibefradil was tested in patients with recurrent high-grade gliomas.

**Table 4:** Clinical trials and drug candidates.

Drug/Strategy	Mechanism of Action	Target Pathway	Clinical Trial Status	Key Findings
Perampanel [29,30,79]	Noncompetitive AMPAR antagonist.	AMPARs neuron–glioma synapse.	PerSurge trial (Phase II); Pilot trials in HGG.	Reduces proliferation, invasion, and TMs formation preclinically. Approved for seizures, safe perioperatively, but maintenance showed limited survival impact.
Sulfasalazine [5,30]	Inhibitor of glutamate release.	xCT antiporter (SLC7A11).	GLUGLIO trial (Phase Ib/II, combination).	Reduces pathological extracellular glutamate levels and excitotoxicity. Monotherapy lacked efficacy in Phase I.
Memantine [6,30]	Noncompetitive NMDAR antagonist.	NMDARs.	GLUGLIO trial (Phase Ib/II, combination).	May prevent synapse formation and increase radiosensitivity. Mild inhibitory effects may offer a better therapeutic window.
Gabapentin/Pregabalin [22,29,30,79]	α2δ-1 subunit binder.	Ca^2+^ channel auxiliary subunit TSP-1 signaling.	GLUGLIO trial (combination).	Reduces synaptogenesis, network synchrony, and proliferation by blocking TSP-1 binding to α2δ-1.
Bumetanide [30]	NKCC1 inhibitor.	NKCC1 Na^+^-K^+^-2Cl^−^ cotransporter GABAergic depolarization.	Preclinical	Counteracts the depolarizing and growth-promoting effect of GABA in DMGs.
Antagonists (e.g., Perphenazine, ONC201) [30]	DRD2 inhibition.	DRD2.	Clinical trials (ONC201) in GBM.	Inactivates oncogenic MET/STAT3 signaling; induces TRAIL ligand-independent apoptosis by activating DR4/5 receptors.
Troriluzole [5,29,30,79]	Glutamate release modulator.	Glutamate reuptake Sodium channels.	GBM AGILE trial (Phase III) for GBM.	Reduces synaptic glutamate by enhancing reuptake and inhibiting release.

## Data Availability

No new data were created or analyzed in this study.

## Abbreviations

The following abbreviations are used in this manuscript:

AMPA	α-amino-3-hydroxy-5-methyl-4-isoxazole propionic acid receptor
AMPAR	AMPA receptor
Akt	Protein kinase B
ASIC1a	Acid-sensing ion channel 1a
BDNF	Brain-derived neurotrophic factor
CaMKII	Calcium/calmodulin-dependent protein kinase II
Cx43	Connexin 43
DMG	Diffuse midline glioma
ECoG	Electrocorticography
EGABA	GABA reversal potential
EGF	Epidermal growth factor
EPSC	Excitatory postsynaptic current
Erk	Extracellular signal-regulated kinase
FGF	Fibroblast growth factor
GAP43	Growth-associated protein 43
GBM	Glioblastoma
GABA	Gamma-aminobutyric acid
GABA_A_R	GABA type A receptor
GCaMP6s	Genetically encoded calcium indicator (variant 6s)
GluR2	Glutamate receptor subunit 2 (GluA2)
HDI-WT	Hemispheric high-grade glioma, IDH-wildtype
IGF-1	Insulin-like growth factor 1
IGF-1R	Insulin-like growth factor 1 receptor
IP3	Inositol 1,4,5-trisphosphate
KCC2	K^+^-Cl^−^ cotransporter 2
LTP	Long-term potentiation
MAPK	Mitogen-activated protein kinase
mGlu3	Metabotropic glutamate receptor 3
mTOR	Mechanistic target of rapamycin
mTORC1	mTOR complex 1
NKCC1	Na^+^-K^+^-2Cl^−^ cotransporter 1
NLGN3	Neuroligin-3
NMDA	N-methyl-D-aspartate (receptor)
OPC	Oligodendrocyte precursor cell
PI3K	Phosphoinositide 3-kinase
PSD95	Postsynaptic density protein 95
PTEN	Phosphatase and tensin homolog
SCLC	Small cell lung carcinoma
SLC7A11	Solute carrier family 7 member 11 (xCT)
SRC	SRC proto-oncogene tyrosine kinase
TME	Tumor microenvironment
TM	Tumor microtube
TrkB	Tropomyosin receptor kinase B (NTRK2)
TSP-1	Thrombospondin-1
VGLUT	Vesicular glutamate transporter
xCT	Cystine/glutamate antiporter (SLC7A11)
